# Job performance and associated factors among nurses working in adult emergency departments at selected public hospitals in Ethiopia: *a facility-based cross-sectional study*

**DOI:** 10.1186/s12912-024-01979-w

**Published:** 2024-05-07

**Authors:** Lema Daba, Lemlem Beza, Merahi Kefyalew, Tilahun Teshager, Fenta Wondimneh, Ashenu Bidiru, Indeshaw Ketema

**Affiliations:** 1 Department of Emergency and Critical Care Nursing, College of Health and Medical Sciences, Haramaya University, Harar, Ethiopia; 2 Department of Emergency Medicine, College of Health Science, Addis Ababa University, Addis Ababa, Ethiopia

**Keywords:** Job performance, Nurses, Emergency department, Addis Ababa, Ethiopia

## Abstract

**Background:**

Optimizing the performance level of nursing staff is crucial for the efficient functioning of hospitals and better patient health outcomes. However, published data on the job performance levels and associated factors of nurses in Ethiopia is limited. Therefore, this study aimed to assess the job performance and associated factors of nurses working in adult emergency departments at selected public hospitals in Addis Ababa, Ethiopia.

**Methods:**

A facility-based cross-sectional study was conducted from March 25 to April 25, 2023, among 172 nurses working in the adult emergency departments of selected public hospitals in Addis Ababa, Ethiopia. A simple random sampling technique was used to select the study participants. Data were collected using pretested, self-administered structured questionnaires. Data were coded, entered into Epi-data version 4.6, and analyzed using Statistical Package for Service Solution (SPSS) Version 27.0.1 software. Data were summarized using descriptive statistics, including mean, frequency, and standard deviation. A binary logistic regression analysis was done to determine factors associated with the performance of nurses. The strength of the association was measured using an adjusted odd ratio (AOR) with a 95% confidence interval (CI), and a *P*-value < 0.05 was considered statistically significant.

**Results:**

The majority of nurses, 70.5% (95% CI: 63.7–77.3), rated their job performance as good. Workload [AOR = 1.70 (95% CI: 1.19–2.44)], remuneration [AOR = 1.89 (95% CI: 1.35–2.67)], rewards [AOR = 1.50 (95% CI: 1.01–2.23)], objectives to be achieved [AOR = 1.88 (95% CI: 1.32–2.67)], and feedback on performance appraisals [AOR = 1.65 (95% CI: 1.17–2.33)] were identified as significantly associated with nurses’ performance.

**Conclusion:**

While the majority of nurses rated their job performance as good, it is important to note that a relevant proportion of nurses rated their job performance as poor. The findings of this study identified that nurses’ performance is influenced by several key factors, including workload, remuneration, rewards, objectives to be achieved, and feedback on performance appraisals. Our findings call for improving nurses’ job performance; therefore, hospitals should consider implementing systems that effectively utilize performance appraisal results and recognize and encourage hardworking nurses.

## Introduction

Job performance refers to how effective healthcare providers are in accomplishing their tasks and responsibilities related to direct patient care, as well as the standard of care provided [1, 2]. The performance of healthcare organizations depends on the knowledge, skills, and motivation of individual employees [3]. The job performance of health workers is a major concern for many healthcare organizations [4].

Nurses are essential human resources in hospitals and primary healthcare settings [5]. Healthcare providers who are competent, motivated, and skilled are the foundation for improved performance in healthcare organizations [6]. Nurses’ performance is a measure of how effectively a nurse fulfills their direct nursing care roles and responsibilities and the quality of care they provide [7, 8]. Nurse performance has a significant impact on the hospital’s competitiveness, patient outcomes, quality of healthcare delivery, and the achievement of organizational goals [4, 5, 9].

Nurses are the largest human resource and work force in the healthcare delivery system worldwide, and their performances have direct impacts on healthcare productivity [10]. Research has shown that nurse performance is influenced by several factors. Knowledge and skills, work experience, education level, competence level, motivation, job satisfaction, work-related stress and burnout, supportive supervision and feedback, training, recognition, work environment, incentives, promotion, remuneration, organizational commitment, and leadership style are among the factors influencing nurses’ performance level [5, 11–20].

The emergency department has a complex structure and multiple factors that influence its performance, and knowing these factors is key to improving the performance of the emergency department [21–23]. Nurses’ performance in the emergency room determines patients’ health outcomes [24, 25]. Nurse underperformance in the emergency department can lead to poor patient outcomes, including longer hospital stays, higher healthcare costs, increased infection risk, and even deaths [5, 26]. Therefore, nurse performance is a priority that must be addressed promptly, as nursing services determine the quality of emergency care [13].

The performance of healthcare workers, including professional nurses, is closely linked to the productivity and quality of care within healthcare organizations [27]. The inadequacy of healthcare providers in the healthcare system places a burden on healthcare providers and reduces individual performance [10, 28, 29]. Hence, assessing the performance level of nurses and its associated factors is of great importance to maintain and even improve the level of care provided for both healthy and sick patients [30].

According to the recent data on nursing posts in Ethiopia, nurses make up the largest number of health workers in the public health sector [5]. This means that the country relies greatly on nurses for service delivery, and their performance is critical for successful healthcare delivery and improved patient health outcomes [5].

In Ethiopia, very few studies were conducted on job performance and associated factors of nurses. Thus, little is known about the job performance levels of nurses and associated factors, Hence, there is a need to assess the job performance and associated factors of nurses for designing evidence-based strategies and improving their job performance. Therefore, this study aimed to determine the job performance level and associated factors of nurses in the adult emergency departments of selected public hospitals in Addis Ababa, Ethiopia. This study provides relevant research-based data on the job performance of nurses that could help policymakers and program directors design evidence-based strategies to improve the level of nurses’ job performance.

## Methods

### Study settings and period

The study was conducted in the emergency departments (EDs) of public hospitals in Addis Ababa, Ethiopia, from March 25 to April 25, 2023. Addis Ababa is the capital city of Ethiopia, which is located in the central part of Ethiopia. There are a total of 12 public hospitals in Addis Ababa [31]. The study was conducted in five randomly selected public hospitals found in Addis Ababa, namely Tikur Anbessa Specialized Hospital (TASH), St. Paulo’s Specialized Hospital, AaBET Hospital, St. Peter’s Referral Hospital, and Alert Referral Hospital.

St. Paul’s Specialized Hospital has 72 nurses working in the adult ED; AaBET Hospital has 98 nurses working in the adult ED; TASH has 42 nurses working in the adult ED; St. Peter’s Referral Hospital has 28 nurses working in the adult ED; and Alert Referral Hospital has 52 nurses working in the adult ED [32]. Hence, a total of 292 nurses were working in the adult EDs of selected public hospitals in Addis Ababa during the survey.

### Study design and population

A facility-based cross-sectional study design was conducted among nurses working in the adult EDs of selected public hospitals in Addis Ababa, Ethiopia. All nurses working in the adult emergency department of each public hospital were invited to take part in the study. Nurses who were on maternity leave, annual leave, sick leave, or severely sick were excluded.

### Sample size determination

The sample size required for the study was determined by using a single population proportion formula considering a 67.8% proportion of nurses’ performance (P) taken from the study conducted at Jimma University Specialized Hospital, Ethiopia [5], a 95% confidence interval, a 5% margin of error, and a 10% non-response rate.

$${\displaystyle \begin{array}{l}\textrm{n}=\frac{{{\left(\textrm{Z}\upalpha /2\right)}^2}^{\ast }\ \textrm{P}\left(1\hbox{-} \textrm{P}\right)}{{\textrm{d}}^2}\\ {}\textrm{n}=\frac{{(1.96)^2}^{\ast }\ 0.678\left(1\hbox{-} 0.678\right)=335}{(0.05)^2}\end{array}}$$

Since the total study population from five selected public hospitals (*N* = 292) is less than 10,000, a correction formula was applied to get the final sample size (nf) as follows.

$${\displaystyle \begin{array}{l}\textrm{nf}=\frac{\textrm{n}}{1+\textrm{n}/\textrm{N}}\\ {}\textrm{nf}=\frac{335}{1+335/292}=156.\end{array}}$$

Then, after adding a 10% non-response rate, the final sample size required for the study was 172.

### Sampling technique and procedures

**Fig. 1:**
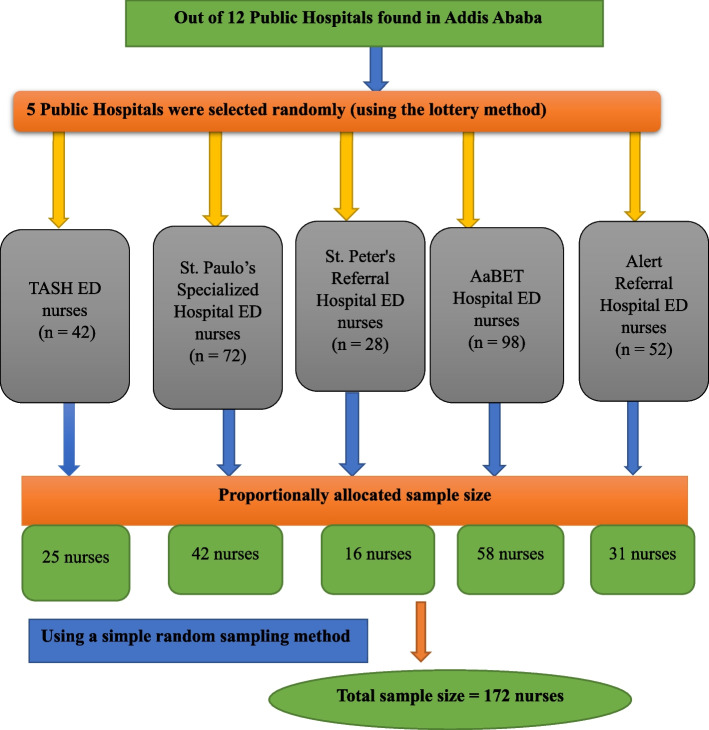
Schematic representation of sampling procedures to select the study participants from selected Public Hospitals in Addis Ababa, Ethiopia, 2023

First, five hospitals (TASH, St. Paul’s Specialized Hospital, AaBET Hospital, St. Peter’s Referral Hospital, and Alert Referral Hospital) were randomly selected (by lottery method) from 12 public hospitals found in Addis Ababa. Then, the total number of nurses working in the adult emergency departments of selected public hospitals was determined by the ward nurse managers. The participants were then allocated from selected five hospitals in proportion to the number of nurses working in the adult ED. Finally, the study participants were selected using a simple random sampling technique (Fig. 1).

### Study variables

The outcome variable for this study was nurses’ job performance. Independent variables include socio-demographic characteristics (age, sex, marital status, education level, work experience), in-service training, resource availability, organizational factors such as feedback, remuneration, recognition, and rewards, and personal factors such as motivation, job satisfaction, interpersonal relationships, knowledge and skills, clinical competency, and related variables.

### Data collection tools and methods

A pretested self-administered structured questionnaire adapted with slight modifications from previous studies [5, 27, 33] was used to collect data on the performance of nurses. The questionnaires consisted of socio-demographic data, performance measurement tool, questions on job satisfaction, and organizational related factors. Nurses’ performance was measured by nine items that are self-rated on a five-point Likert scale (1 = very poor, 2 = poor, 3 = good, 4 = very good, and 5 = excellent). The scores were averaged so as to show each participant’s job performance level, ranging from 9 to 45 [27, 33]. Respondents were categorized as having “good job performance” if their average job performance score was greater than or equal to the computed overall mean value of job performance (mean ≥ 3.56), and “poor job performance” if their average job performance score was less than the computed overall mean value of job performance (mean < 3.56) [33, 34]. The Cronbach alpha coefficient value was 0.72, showing the performance measure was reliable. Nurses’ Job satisfaction was measured by eleven items on a five-point Likert scale (1 = strongly dissatisfied, 2 = dissatisfied, 3 = average, 4 = satisfied, and 5 = very satisfied). The scores were averaged so as to show each respondent’s satisfaction level, ranging from 11 to 55. Respondents with an average score less than the mean value were classified as dissatisfied, and those with an average score of the mean value and above were considered satisfied. The Cronbach alpha coefficient was 0.839, showing the job satisfaction measure was reliable. Organizational related factors were measured by eight items using a Likert scale with five responses (1 = strongly disagree, 2 = disagree, 3 = neutral, 4 = agree, and 5 = strongly agree). Data were collected by trained data collectors and supervisors using pretested self-administered questionnaires.

### Data quality assurance

A pretested and validated self-administered structured data collection tool was used to ensure data quality. Two days training were given to the data collectors and supervisors on the objectives of the study, the contents of the data collection tool, and other related issues. A pretest was done on 10% of the sample size at Yekatit 12 Referral Hospital before the actual data collection period to check for the reliability and validity of the data collection tools. The questionnaires were reviewed and checked for incompleteness and inconsistency, and necessary amendments were made based on the pretest results. The collected data were carefully checked for completeness, accuracy, and consistency by supervisors and the principal investigator on a daily basis.

### Data processing and analysis

The collected data were cleaned and checked for completeness and consistency, coded and entered into Epi-data version 4.6, and analyzed using SPSS Version 27.0.1 software. Descriptive statistics such as frequency, mean, and standard deviation were used to summarize the data. A binary logistic regression model was used to examine factors predicting the level of nurses’ performance. Variables with a *P*-value of less than 0.25 in the bivariable logistic analysis were entered into the multivariable logistic analysis to determine factors significantly associated with the performance level of nurses. The logistic regression goodness of fit of the model was checked using the Hosmer and Lemeshow test and showed a good fit at a P-value of 0.985. An adjusted odd ratio with a 95% CI was computed to measure the strength of the association. A P-value < 0.05 was used to declare statistical significance. Finally, the study results were displayed using tables and figures and presented with narrative descriptions.

### Ethical considerations

The study was conducted following the principles of the Helsinki Declaration. Ethical clearance was obtained from Addis Ababa University’s, College of Health Science’s Institutional Health Research and Ethics Review Committee (IHRERC) under Ref. No. PM 23/661. After ethics approval, a written official letter of cooperation was submitted to each hospital before the commencement of the data collection to obtain administrative permission. Informed voluntary, written, and signed consent was obtained from the study participants after they were informed of the aim, purpose, and benefits of the study. The confidentiality of the information was ensured throughout the data collection and dissemination processes.

## Results

### Socio-demographic characteristics

A total of 166 study participants completed and returned the questionnaires, yielding a response rate of 96.5%. The mean (±SD) age of the study participants was 29 (±6.23) years. Among the respondents, the majority (58.4%) were aged 26 to 33 years, more than half (57.8%) were female, and 85 (51.2%) were single in marital status. Regarding their education status, more than two-thirds (64.5%) of the respondents had a BSc in Nursing, and 51 (30.7%) of them had 2–3 years of work experience in the emergency departments (Table 1).

**Table 1:** Socio-demographic characteristics of the study participants in the adult EDs of selected public hospitals in Addis Ababa, Ethiopia, 2023 (*n* = 166)

Variables	Category	Frequency (***N***)	Percentage (%)
**Age (in years)**	18–25	29	17.5
	26–33	97	58.4
	34–40	40	24.1
**Sex**	Male	70	42.2
	Female	96	57.8
**Marital status**	Married	74	44.6
	Single	85	51.2
	Others^a^	7	4.2
**Level of qualifications**	BSc in Nursing	107	64.5
	MSc in Nursing	59	35.5
**Monthly income (Eth. birr)**	3000–6000	45	27.1
	6001–8000	72	43.4
	8001–10,000	43	25.9
	> 10,000	6	3.6
**Work experience at the emergency department (in years)**	< 2	44	26.5
	2–3	51	30.7
	4–5	43	25.9
	> 5	28	16.9

^a^Widowed, Divorced/Separated

### Nurses’ performance

**Fig. 2:**
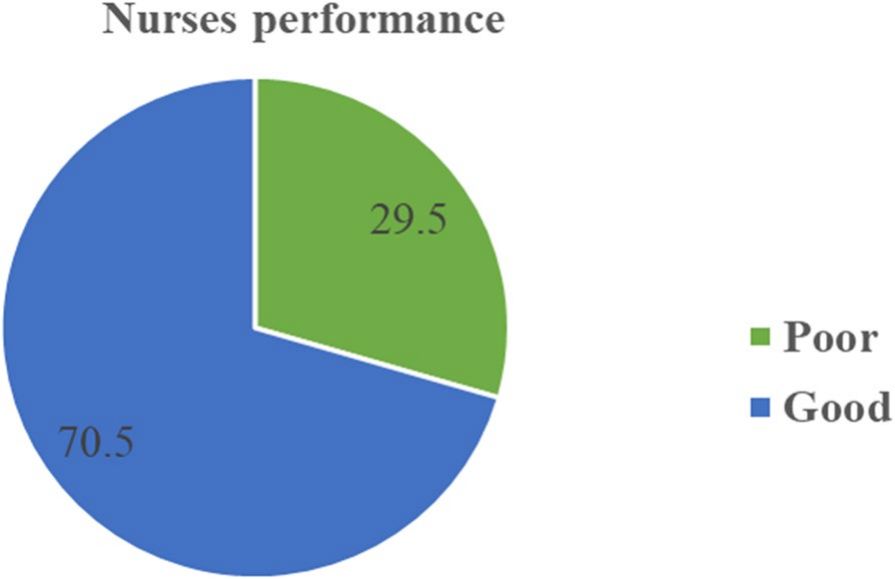
Overall self-rated job performance of nurses in the adult EDs of selected public hospitals in Addis Ababa, Ethiopia, 2023 (*n* = 166)

The self-rated performance assessment of nurses was calculated using total measures of performance assessment items such as sick and emergency leave, attendance and punctuality, optimizing personal skills, relationships with patients and coworkers, quality of work, relationships with supervisors, improving work methods, and overall performance compared to co-workers. The overall self-rated job performance of nurses was 70.5% (95% CI: 63.7–77.3). The overall self-rated job performance showed that the majority of nurses, 117 (70.5%), working in the adult EDs of selected public hospitals in Addis Ababa had good job performance, while 49 (29.5%) had poor job performance (Fig. 2).

### Knowledge and skill

The majority of respondents rated their knowledge and skills satisfactory, as indicated by the mean score of different items that assessed their knowledge and skills. About 79.6% of respondents thought that they had good interpersonal relationship skills, with a mean score of 3.98, followed by time management (78.6%), applying a nursing care plan (78.2%), and work quality (77.8%). The respondents rated themselves as having an average level of knowledge and skills in providing health education (72.8%) and relationships with their supervisors (72.2%) (Table 2).

**Table 2:** Mean score of self-rated knowledge and skill items by nurses working in the adult EDs of selected public hospitals in Addis Ababa, Ethiopia, 2023 (*n* = 166)

Knowledge and Skill	Mean	Std. Deviation
Planning of nurse care	3.84	0.96
Relationship with your supervisors	3.61	1.164
Relationship with your colleagues	3.84	0.949
Improving work methods	3.72	1.11
Overall performance as compared to your coworkers	3.74	1.15
Implementation of nursing care plans	3.91	1.03
Assessment of patient	3.73	0.97
Implementing nursing performance standard	3.75	1.09
Providing health education for patient and family	3.64	1.16
Clinical competency	3.72	1.12
Interpersonal relation	3.98	0.89
Patient counselling skills	3.83	0.97
Self-evaluation in terms of outcome performance	3.72	1.03
Supervision of nursing care	3.86	0.82
In-service training	3.01	1.22
Management of time	3.93	0.79
Care of facilities, equipment, and supplies	3.78	0.96
Optimization of quality care	3.86	0.86

### Performance appraisal and its utilization

**Fig. 3:**
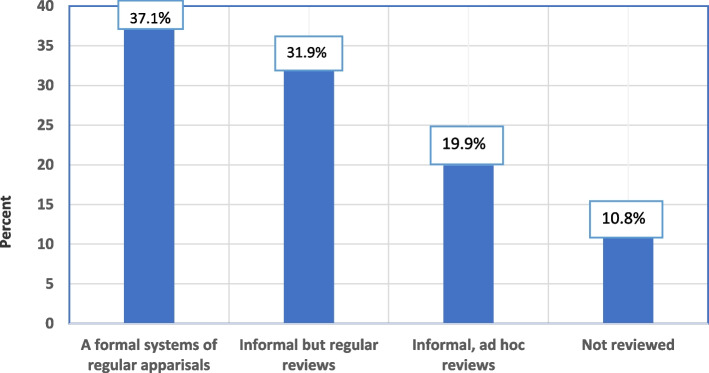
Method of performance appraisal of nurses in the adult EDs of selected public hospitals in Addis Ababa, Ethiopia, 2023 (*n* = 166)

**Fig. 4:**
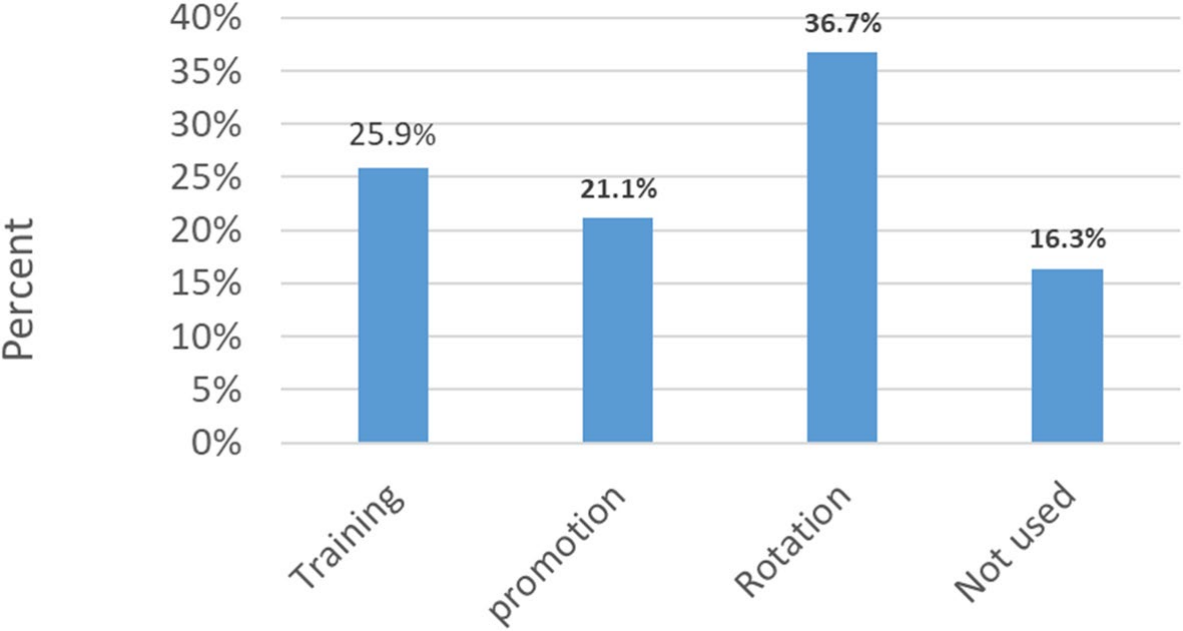
Utilization of the results of performance appraisal by selected public hospitals in Addis Ababa, Ethiopia, 2023 (*n* = 166)

Sixty-two (37.3%) of the respondents reported that their performance was reviewed on a regular schedule and under a formal method of appraisal, which included a review of prior performance and the setting of objectives; 53 (31.9%) stated that their performance was reviewed informally, but with regular assessments that included a discussion of past performance and agreed with future action; and 33 (19.9%) reported that their performance was reviewed informally with a specific review when there was a performance problem (Fig. 3). The majority of respondents, 43 (25.9%), 61 (36.7%), and 35 (21.1%), said the results of performance appraisals were used for training, rotation, and promotion, respectively (Fig. 4).

### Performance management system

The mean score of different items showed that the majority of respondents were not satisfied with the performance appraisal system and its utilization. The majority, 84 (50.6%) and 75 (45.2%), of them were uncertain with performance standards expected from staff being clear and understood by all and objectives to be achieved known by individuals, with mean scores of 3.27 and 3.23, respectively. Seventy-eight (47.6%) disagreed that feedback on how staff were performing was provided throughout the year, with a mean score of 2.97. While 48 (28.9%) of them disagreed with staff that they were given the opportunity to comment on the result of their performance, with a mean score of 2.81 (Table 3).

**Table 3:** Performance appraisal and its utilization system by selected public hospitals in Addis Ababa, Ethiopia, 2023 (*n* = 166)

Performance appraisal and utilization	Mean	Std. Deviation
Objectives to be achieved are known by individuals to be assessed	3.23	1.157
Performance standards expected from staff are clear and understood by all	3.27	1.215
Regular constructive feedback on performance appraisal results	3.05	1.156
Feedback on how the staff is performing is provided throughout the year	2.97	1.145
Prompt action is taken when performance falls below accepted standards	3.03	1.151
My manager’s supervision inspires me to do my best	2.83	1.327
Staff are given the opportunity to make comments on the result of their performance	2.81	1.347

### Remuneration, benefits and recognition

Most of the nurses working in the adult EDs of selected public hospitals in Addis Ababa were not satisfied with the remuneration, benefits, and recognition they received from the institution. The majority of them disagreed that remuneration was competitive with other similar organizations and given according to their responsibility, with mean scores of 3.25 and 3.04, respectively. Sixty-one (36.8%) were unaware of their fringe benefits, and almost half (49.4%) disagreed that hardworking nurses were being recognized, with mean scores of 3.07 and 2.78, respectively. Similarly, 54 (32.6%) of nurses disagreed with the statement that remuneration was based on their level of experience, with a mean score of 3.19, and 67 (40.4%) disagreed with the statement that there is an opportunity for career advancement, with a mean score of 2.95 (Table 4).

**Table 4:** Remuneration, benefits, and recognition of nurses working in the adult EDs of selected public hospitals in Addis Ababa, Ethiopia, 2023 (*n* = 166)

Remuneration, benefits and recognition	Mean	Std. Deviation
Remuneration is competitive compared to other similar organizations	3.25	1.254
Remuneration is based on the level of experience	3.19	1.268
Remuneration is according to your job responsibilities	3.04	1.208
You are aware of the fringe benefit	3.07	1.260
Opportunities exist for your career development	2.95	1.180
Nurses who work hard are rewarded	2.78	1.380

### Staffing and work schedule

The results of this study indicated that almost half (49.4%) of the respondents disagreed with the fairness of the overall work schedule with a mean score of 2.73, and most respondents disagreed that they had the opportunity to make inputs into staffing policies and procedures with a mean score of 2.66. The majority (62.1%) of the respondents disagreed that the allocated staff in their unit are adequate to cover the current workload, with a mean score of 2.55 (Table 5).

**Table 5:** Staffing and work schedule system of selected public hospitals in Addis Ababa, Ethiopia, 2023 (*n* = 166)

Staffing and work schedule	Mean	Std. Deviation
Opportunity to provide feedback on staffing policies & procedures exist	2.66	1.195
Flexible work schedule is available	2.86	1.260
The overall work schedule is acceptable	2.73	1.208
The overtime work is acceptable	3.56	0.987
Good balance between people who supervise work and those who do it	3.22	1.241
The staff in my unit is adequate to handle the current workload	2.55	1.228
Staff care and support in the form of counselling are available	3.16	1.073

### Staff development

The study showed that more than half (61.5%) of the respondents were dissatisfied with job-specific refresher courses, and the in-service training filled the skill gap with mean scores of 2.45 and 2.54, respectively. Half (50%) and 90 (54.2%) of respondents disagreed with the opportunity for advancing in the organization and the availability of opportunities for continuing education, with mean scores of 3.26 and 3.10, respectively. The majority (75.5%) felt that they were not given the necessary training to ensure job effectiveness, with a mean score of 2.62 (Table 6).

**Table 6:** Nurses’ perceptions concerning staff development strategies of selected public hospitals in Addis Ababa, Ethiopia, 2023 (*n* = 166)

Staff development	Mean	Std. Deviation
Opportunities for promotion within the institutions are available	3.26	1.084
Good chances for further education are available	3.10	1.126
Training is provided to ensure job effectiveness	2.69	1.239
Job-focused refresher courses are available	2.45	1.110
In-service training adequately fills the skill gap	2.54	1.126
Incompetent nurses are identified and given the necessary support	2.58	1.145
Effective leadership/management training available	2.62	1.183
Nurses help to identify their staff development needs	2.72	1.179

### Workspace and environment

Nurses working in the adult EDs of selected public hospitals in Addis Ababa were dissatisfied with the workspace and environment. Respondents disagreed with the availability of necessary instruments, materials and supplements, working instruments, guidelines for infection control strategy, and whether the work environment is safe and free from environmental hazards, with mean scores of 2.77, 2.43, 2.74, 2.83, and 2.59, respectively (Table 7).

**Table 7:** Nurses’ perceptions regarding the work environment of selected public hospitals in Addis Ababa, Ethiopia, 2023 (*n* = 166)

Workspace and environment	Mean	Std. Deviation
My workplace is safe and free of environmental hazards	2.59	1.033
Good layout for working play	2.95	1.172
Comfortable temperature	3.35	1.100
Necessary instruments are available	2.77	1.122
Working instrument available	2.74	1.221
Material and supplements are sufficient	2.43	1.188
Antiseptic hand solutions are available to protect staff and patients	3.05	1.288
Guideline for infection control strategy is available	2.83	1.196

### Factors associated with nurses’ performance

In the multivariable logistic analysis, workload, remuneration, rewards, objectives to be achieved, and feedback on performance appraisals were identified as significantly associated with nurses’ performance. Nurses who had a light workload were 1.7 times [AOR = 1.70 (95% CI: 1.19–2.44)] more likely to have good performance than those who had a heavy workload. Nurses who had good remuneration were 1.89 times [AOR = 1.89 (95% CI: 1.35–2.67)] more likely to have good performance than nurses who had poor remuneration. Nurses who had rewards for their work were 1.5 times [AOR = 1.50 (95% CI: 1.01–2.23)] more likely to have good performance than nurses who did not. Moreover, nurses who knew the objectives to be achieved were 1.88 times [AOR = 1.88 (95% CI: 1.32–2.67)] more likely to have good performance than nurses who did not. Nurses who perceived feedback on performance appraisals were 1.65 times [AOR = 1.65 (95% CI: 1.17–2.33)] more likely to have good performance than nurses who did not (Table 8).

**Table 8:** Factors associated with nurses’ performance level in the adult EDs of selected public hospitals in Addis Ababa, Ethiopia, 2023 (*n* = 166)

Variables	Category	Nurses’ Performance		COR (95% CI)	AOR (95% CI)	***P***-value
		Poor = 49	Good = 117			
		***N*** (%)	***N*** (%)			
**The allocated staff sufficient to cover the current workload**	Disagree	37 (35.92)	66 (64.08)	1	1	
	Agree	12 (19.05)	51 (80.95)	1.81 (1.35,2.43)	1.70 (1.19,2.44)	0.004*
**Clinical competence**	Poor	24 (53.33)	21 (46.67)	1	1	
	Good	25 (20.66)	96 (79.34)	3.39 (2.11,7.13)	2.08 (0.96,6.89)	0.082
**In-service training**	Poor	36 (73.5)	57 (48.7)	1	1	
	Good	13 (26.5)	60 (51.3)	2.92 (1.40,6.05)	1.11 (0.34,7.36)	0.063
**Remuneration is according to experience**	Disagree	28 (51.85)	26 (48.15)	1	1	
	Agree	21 (18.75)	91 (81.25)	2.19 (1.53,2.72)	1.89 (1.35,2.67)	0.001*
**I find my work rewarding**	Disagree	31 (37.80)	51 (62.20)	1	1	
	Agree	18 (21.43)	66 (78.57)	2.08 (1.01,1.87)	1.50 (1.01,2.23)	0.044*
**Objectives to be achieved are known by individuals to be assessed**	Disagree	34 (45.33)	41 (54.67)	1	1	
	Agree	15 (16.48)	76 (83.52)	4.12 (1.43,2.52)	1.88 (1.32,2.67)	0.001*
**Feedback on how the staff is performing is provided throughout the year**	Disagree	28 (35.89)	50 (64.11)	1	1	
	Agree	21 (23.86)	67 (76.14)	1.76 (1.31,2.33)	1.65 (1.17,2.33)	0.004*

*Significantly associated variables at a *P*-value < 0.05

## Discussion

Healthcare providers’ job performance level is a cornerstone for the productivity of healthcare organizations [6]. Optimal performance levels of nurses are critical for the productivity of healthcare organizations, while underperformance reduces hospital competitiveness and productivity and contributes to poor patient health outcomes [8, 26]. This study assessed nurses’ performance levels and identified factors affecting their performance.

In the current study, the overall self-rated job performance of nurses was 70.5% (95% CI: 63.7–77.3). This indicates that the majority of nurses had good job performance. The result of this study is consistent with the findings of the studies from Jimma, Ethiopia (67.8%) [5], Bahir Dar, Ethiopia (76.64%) [35], and Medan, Indonesia (72%) [11]. This could be due to the similar study design (cross-sectional), study subjects (nurses), and study settings (hospitals). In contrast, this is higher than the findings of the study done at Datu Beru Takengon General Hospital, Indonesia (41.5%) [8]. This discrepancy might be due to different study settings, the difference in education status (the majority of nurses had a BSc degree and above in this study, while most of the nurses from Datu Beru Hospital were diploma level III), and various levels of hospitals.

The majority of nurses in the adult EDs of selected public hospitals in Addis Ababa reported that their performance was regularly evaluated in both formal and informal ways. This is in line with the findings of the studies conducted in Jimma, Ethiopia [5], and Namibia [27]. However, most nurses were not satisfied with the utilization of performance appraisal results. The majority of them reported that they were not given the chance to comment on their performance results, which is similar to the findings of the study done in Jimma, Ethiopia [5]. This could lead nurses to continue poor exercise, which can have a negative impact on patient outcomes.

Most nurses working in adult EDs in selected public hospitals in Addis Ababa were not satisfied with the remuneration, benefits, and recognition they received from the institution. The majority of them reported that their remuneration was not according to their level of experience. Most of the nurses also reported dissatisfaction with fringe benefits and disagreement with hardworking nurses being recognized. In addition, almost half of the nurses stated there was a problem with the fairness of the work schedule and disagreed with the opportunity to make inputs into staffing policies and procedures. This is in line with the findings from the studies done in Jimma, Ethiopia [5], and Namibia [27]. This could be attributed to a decrease in nurses’ motivation to work.

According to the findings of this study, workload, remuneration, rewards, objectives to be achieved, and feedback on performance appraisals were significant predictors of nurses’ performance. The workload had a significant statistical association with nurses’ performance. Nurses with a light workload were 1.7 times more likely to perform well than nurses with a heavy workload. This is in line with the findings of the studies conducted in Indonesia [8, 11, 36], Pakistan [37], and Iraq [38]. This might be due to insufficient staff, which leads nurses to have more workload and directly affects nurses’ performance.

The findings of this study revealed that nurses who had good remuneration were almost two times more likely to have good performance than nurses who did not. This is consistent with the findings of the studies from the Philippines [25] and Uganda [39]. This suggests that good remuneration enhances nurses’ job performance by increasing their motivation. However, in the study from Jimma, Ethiopia, remuneration had no statistical association with nurses’ performance [5]. This discrepancy might be due to differences in time and place.

The findings of this study also showed that nurses who had received good rewards for their work were 1.5 times more likely to have good performance than nurses who did not. This finding is supported by the findings of the studies conducted in Egypt [13], Iraq [38], Ghana [40], and Indonesia [41], showing that rewarding nurses is positively associated with their performance. This suggests that nurses who received rewards had a higher chance of performing well in their jobs than nurses who did not.

The findings of this study further showed that the objective to be achieved was a significant predictor of nurses’ performance. Nurses who knew the objectives to be achieved were almost two times more likely to have good performance than nurses who did not. This finding is supported by the findings of the study conducted in Namibia [27]. This is justified by the fact that even if nurses have knowledge and training, their performance can be dramatically affected if they don’t know the objectives to be achieved.

Furthermore, nurses who perceived feedback on performance appraisals were 1.65 times more likely to have good performance compared to nurses who did not. This finding is supported by the findings of the studies from Jimma, Ethiopia [5], Egypt [13], Namibia [27], and Sorong District Hospital [42]. This indicates that feedback on performance appraisals had a significant impact on nurses’ performance because the frequent feedback they receive allows them to identify their strengths and weaknesses, which can help them perform their activity in a proper manner and improve their job performance.

### Strengths and limitations

This study provides relevant research-based data on nurses’ performance levels and identifies factors that influence their performance, which could help stakeholders design evidence-based interventions. This study had the following limitations: First, due to the nature of the cross-sectional study, it was difficult to establish the cause-effect relationship between variables. Second, the small sample size used in the study may not represent a larger population. In addition, respondents may have been biased in their self-reports of job performance. Another limitation of this study is that an observation checklist was not used. This study asks respondents about their perceived knowledge and skills, and measuring performance through self-report is not the best way to gather information. The study did not include primary healthcare facilities and private clinics, so no comparison was made between public and private clinics, which may also be a limitation of this study.

## Conclusion

Even though the majority of nurses rated their job performance as good, it is important to note that a relevant proportion of nurses rated their job performance as poor. The findings of this study identified workload, remuneration, rewards, objectives to be achieved, and feedback on performance appraisals as independent predictors of nurses’ performance. Therefore, to improve nurses’ job performance, hospitals should consider implementing systems that effectively utilize performance appraisal results, as well as recognizing and encouraging hardworking nurses. In addition, a further study assessing the job performance of nurses and associated factors that uses observation checklist and large sample size is recommended to fill the gap in the study.

## Abbreviations

AaBET: Addis Ababa Burn Emergency and Trauma Center
AO: Adjusted Odd Ratio
CI: Confidence Interval
COR: Crude Odd Ratio
ED: Emergency Department
SPSS: Statistical Package for Service Solution
TASH: Tikur Anbessa Specialized Hospital
